# Metabolic Profile and Performance Responses of *Ranunculus asiaticus* L. Hybrids as Affected by Light Quality of Photoperiodic Lighting

**DOI:** 10.3389/fpls.2020.597823

**Published:** 2020-11-19

**Authors:** Petronia Carillo, Emilia Dell’Aversana, Giuseppe Carlo Modarelli, Giovanna Marta Fusco, Stefania De Pascale, Roberta Paradiso

**Affiliations:** ^1^Department of Environmental, Biological and Pharmaceutical Sciences and Technologies, University of Campania Luigi Vanvitelli, Caserta, Italy; ^2^Department of Agricultural Sciences, University of Naples Federico II, Portici, Italy

**Keywords:** geophytes, tuberous roots, photosynthetic carbohydrates, amino acids, proteins, alanine, GABA

## Abstract

*Ranunculus asiaticus* is a quantitative long day plant grown for cut flowers and flowering potted plants production. We evaluated the influence of light spectrum of three light sources for end-of-day photoperiodic treatments, with different phytochrome photoequilibria (PPE) induced at plant level, on the metabolic profiling of two hybrids of *R. asiaticus* L., MBO and MDR, in plants from vernalized tuberous roots. The following treatments were compared with natural day length (NL): white fluorescence lamp (FL, PPE 0.84), light emitting diodes (LEDs) Red:Far Red light at 3:1 ratio (R:FR 3:1, PPE 0.84), and LEDs Red:Far Red light at 1:3 ratio (R:FR 1:3, PPE 0.63). Measurements were carried out to evaluate the time course of carbohydrate, amino acid, and protein levels throughout the growing cycle in tuberous roots and leaves, in relation to the different plant stages (pre-planting, vegetative phase, and flowering). The study of metabolic profiling suggested that the differences between the tuberous root reserves of the two *R. asiaticus* hybrids could be responsible for the capacity of MBO to exert an early flowering. In particular, the proton-consuming synthesis during the pre-planting of two amino acids, alanine and γ-*aminobutyric acid* (GABA), is able to buffer the cytoplasmic acidosis and pH altered by the vernalization process, and GABA itself can efficiently scavenge reactive oxygen species. This fast response to the stress caused by vernalization allows MBO plants to accelerate the process of vegetative development and flowering. Some other changes in metabolites profile were certainly related to the different responses to day length and photoperiodic light quality in the two hybrids, such as dose exerted by low R:FR lighting in both MBO and MDR. However, most of the responses are under a strict genetic control.

## Introduction

*Ranunculus asiaticus* L. (family Ranunculaceae) is an a perennial geophytesgeophyte, native of the Mediterranean basin and Asia Minor, grown as annual crop for flower stems and flowering potted plants production (De Hertogh, 1996). Cultivation of *R. asiaticus* has been rising during the last years all over the world, also thanks to the breeding and the development of many hybrids (Beruto et al., 2018).

In the natural Mediterranean habitat, dormant tuberous roots sprout in autumn, when the first rain rehydrates the dried tissue, and develop a rosette of long petiole leaves, plants flower from February to May, and then enter in dormancy, and the aerial part wilt and disappear during summer (Horovitz, 1985; Meynet, 1993). As many spring flowering geophytes exhibit a summer rest period, *R. asiaticus* requires a warm - -cold - -warm sequence to express active growth and complete its life cycle (Le Nard and De Hertogh, 1993).

Flowering earliness and flower stem production and characteristics (i.e., stem length and flower size) vary widely with the plant genotype, the size of tuberous roots and the procedure of storing and preparation before planting, and the growing conditions. In general, plants from bigger roots show an earlier floral induction and produce more flowers compared to than from smaller roots, due to the greater quantity of reserve starch available for flower stem development (Meynet, 1993).

For production of propagation material, the tuberous roots harvested after plant wilting are dehydrated to less than 15% of moisture content (Meynet, 1993). During growth, plants exhibit a low temperature requirement (night/day regime 5–10/12–25°C, optimum day 16°C) and a medium to high light intensity requirement. Referring to photoperiodic requirements for flowering, *R. asiaticus* is classified as a quantitative long day (LD) plant (Horovitz, 1985). Flowering is influenced by both the thermal history of tuberous roots and the photoperiod during plant growth. Particularly, cold treatments of tuberous roots (vernalization) anticipate sprouting, leaf rosette formation, and flowering (Beruto et al., 2009); therefore, the use of vernalized propagation material is a common practice for production scheduling of cut flowers in ranunculus, similarly, to many flower geophytes.

It is known that the exposure to low temperature promotes the overcoming of bulb dormancy in *Lilium pumilum* by promoting starch degradation and increasing sucrose content and availability (Wang et al., 2018). Besides, since bulb dormancy is regulated by the abscisic acid (ABA) and gibberellic acid (GA) balance (Liu et al., 2011), vernalization releases dormancy by activating the metabolic pathways involved in the downregulation of ABA and priming the biosynthesis of GA active forms (Wang et al., 2018). However, recently, we found that vernalization not only induced a faster hydrolysis of starch in *R. asiaticus* tuberous roots but also promoted a strong accumulation of hexoses and proline, acting as compatible osmolytes and reactive oxygen species (ROS) scavengers (Carillo et al., 2019a). Moreover, minor amino acids, whose accumulation is correlated with that of hexoses under stress (Fritz et al., 2006), can act as antioxidants and/or as alternative electron donors for the mitochondrial electron transport chain, accelerating plant metabolism and flowering (Woodrow et al., 2017; Carillo, 2018).

The influence of light spectrum on plant growth, photosynthesis, and photomorphogenetic responses, including flowering, is well documented (Devlin et al., 2007; Ouzounis et al., 2015; Dueck et al., 2016). Among the plant photoreceptors able to perceive the different light wavelengths to regulate plant development and shaping and metabolism, phytochromes are appointed for the absorption of red (R, 600–700 nm) and far red (FR, 700–800 nm) light. They are present in all plant tissues in two forms with different absorption properties: phytochrome red (Pr, absorption peak at 660 nm) and phytochrome far red (Pfr, absorption peak at 730 nm). R light converts Pr to Pfr, whereas FR light has the opposite effect; hence, changes in spectral light composition influence the ratio between the two forms and the phytochrome photoequilibria (PPE = Pfr/Ptot) (Sager et al., 1988) involved in several physiological functions, such as seed germination, flowering, tuberization, bud dormancy, and shade-avoidance responses (Fukuda, 2019).

Red and Far Red light are perceived at very low light intensity, so that the threshold irradiance in photoperiodic lighting effective to promote flower induction in some herbaceous species is between 0.05 and 0.40 μmol m^–2^ s^–1^ (Whitman et al., 1998). Accordingly, a light intensity between 1 and 3 μmol m^–2^ s^–1^ is adopted by growers for photoperiodic treatments (Zhang and Runkle, 2019).

The importance of the PPE value induced at plant level by R and FR light in the regulation of the flowering process of LD plants has been deepened recently, thanks to the use of light emitting diodes (LEDs) (Craig and Runkle, 2016; Dueck et al., 2016). It has been demonstrated that the light quality of the different light sources for greenhouse photoperiodic lighting affects flowering of LD plants by influencing the PPE in plant. However, light requirement in terms of intensity and quality can vary among the species, and the best light spectrum to promote flowering is known for only a few flower crops. Specifically, R and FR proportion creating an intermediate PPE (0.63–0.80) has been proven to be more effective in some LD species (*Antirrhinum majus*, *Fuchsia* × *hybrida*, *Petunia* × *hybrida*, *Rudbeckia hirta*) than ratios inducing higher PPE (above 0.80) (Craig and Runkle, 2016). Conversely, results obtained in our experiment on *R. asiaticus* in plants from rehydrated and vernalized tuberous roots revealed a stronger advance of flowering under LEDs light with 3:1 R:FR ratio (estimated PPE 0.84) than with 1:3 ratio (estimated PPE 0.63) and highlighted a hybrid specific response (Modarelli et al., 2020b).

As known, underground storage organs in geophytes are modified roots or stems that evolved to survive adverse environmental conditions. Little is known about storage organs composition and metabolism during their rest period in nature or storage in commercial practice. Accumulation of large amounts of reserve carbohydrates in these organs is critical to ensure a supply of carbon and energy for their maintenance during unfavorable conditions and for rapid vegetative growth when sprouting can occur. Starch is the most abundant reserve carbohydrate in geophytes, however, other carbohydrates (e.g., fructan and glucomannan) can be synthesized in place of or in addition to starch (Ranwala and Miller, 2008), and the carbohydrate composition may differ among species (Miller, 1992). Despite the importance of storage carbohydrate metabolism in ornamental geophytes and their worldwide economic significance, information about their identity and distribution among species is scarce, and most importantly, data on source–sink relationships between storage organs and above-ground plant parts are limited to a few major species. In principle, carbohydrates stored in the under-ground parts are mobilized during resprouting, acting as the major supply of carbon for the early stages of regrowth; accordingly, the carbohydrate content of storage organs varies from time to time and depends on environmental conditions (Addai, 2010). In general, during the early shoot growth of most geophytes (e.g., tulip), when stored reserves are utilized, starch content decreases and subsequently increases after anthesis, and at this point, carbohydrate filling in under-ground organs is rapid. In tulip, low temperature is essential for the mobilization of reserves and the accumulation in bulb scales of soluble constituents, available to be transported into the shoots for elongation and growth (Ohyama et al., 1998). As a consequence, dry weight (DW) of the mother bulb decreases gradually after planting and during sprouting until anthesis.

Conversely to other geophytes or wild ranunculus species (Madsen, 1993; Madsen and Brik, 1997), little is known about the plant metabolism in *R. asiaticus*, and no study seems to be available on the influence of photoperiodic lighting on plant physiology, apart from our recent experiments (Modarelli et al., 2020a,b).

We investigated the influence of light spectrum of three light sources for photoperiodic lighting, inducing different PPE at plant level, in two hybrids of *R. asiaticus* with a different flowering earliness, in plants from rehydrated and vernalized tuberous roots grown in unheated glasshouse. The effects of artificial lighting and of interactions between plant genotype and light duration and quality were studied on leaf net photosynthesis, plant growth, flowering earliness, and duration and metabolic profile. We reported the results of this experiment for photosynthesis, growth, and flowering in Modarelli et al. (2020a). In the same paper, these results were linked to the metabolic profile (starch, soluble sugars, soluble proteins, amino acids, and polyphenols) of only leaves at the sole flowering phase. In the present paper, we show the time course of the above-mentioned metabolites throughout the whole growing cycle in both tuberous roots and leaves, in relation to the different phenological phases (pre-planting, vegetative phase, and flowering). This in-depth analysis aimed to unveil the importance of root–shoot relationship in determining the differences of plant behavior between different genotypes and of plant sensitivity to light environment. To our best knowledge, our results provide the first comprehensive analysis of metabolism of the whole plant, including both storage organ and leaves, in *R. asiaticus*.

## Materials and Methods

The experiment was carried out in an unheated greenhouse at the Department of Agricultural Sciences of the University of Naples Federico II, located in Portici (Naples, Italy—40°49′N, 14°20′E), from the middle of November 2018 until the end of March 2019.

### Plant Materials and Culture

Plants of the hybrids MBO (early flowering) and MDR (medium earliness) (Biancheri Creazioni, Italy)^1^ were obtained from tuberous roots of the most common size for each hybrid (3–4 and 4–5 cm, respectively). Dry tuberous roots were subjected to rehydration, followed by vernalization, through exposure at 3.5°C for 2 weeks and then pre-sprouted at 12°C for 2 weeks. Plants were grown in a pot on a mixture of perlite and peat (70:30 in vol.).

The mean values of air temperature and relative humidity (day/night) recorded during the experiment (125 days) were 21.0 ± 3.0/9.8 ± 1.9°C and 59.9 ± 10.7/71.4 ± 21.7%, respectively (hourly measurements; mean value ± standard deviation).

Irrigation was alternated with fertigation (4 pulses per week in total). In the nutrient solution (recipe Hoagland full strength), pH and electrical conductivity were measured with a portable pH–EC multi-parameter sensor (HI9813 series; Hanna Instruments Intl.) and kept at 5.5 and 1.7 dSm^–1^, respectively.

### Light Sources and Photoperiodic Treatments

Photoperiodic lighting was applied as end-of-day (EOD) lighting treatment to extend the day length to 14 h (critical photoperiod for *R. asiaticus* L.) starting from sunset, from December 10 (24 days after planting), when all the tuberous roots were fully sprouted, until the end of the experiment. The duration of lighting treatment to reach 14 h day length was calculated weekly, on the basis of the natural photoperiod, and ranged between approximately 4 h and 30 min in December and about 2 h in March. Four photoperiodic treatments were compared:

– NL: natural day length, ranging from 9:16 (2nd week of December) to 11:54 h (3rd week of March);
– FL: NL + EOD lighting with a compact white fluorescence lamp (Phillips Master 13W, color temperature 2,700°K), estimated PPE 0.84;
– R:FR 3:1: NL + EOD lighting with monochromatic R and FR LEDs at 3:1 ratio (emission peak at 662 and 743 nm, respectively), estimated PPE 0.84;
– R:FR 1:3: NL + EOD lighting with monochromatic R and FR LEDs at 1:3 ratio (emission peak at 662 and 743 nm, respectively), estimated PPE 0.63.

**FIGURE 1:**
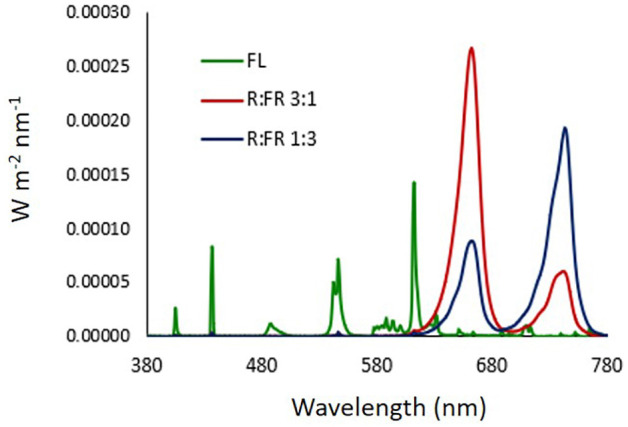
Light emission spectra of light sources. FL: compact white fluorescence lamp (Phillips Master 13W, color temperature 2,700°K); R:FR 3:1: monochromatic red and far red LEDs at ratio 3:1; R:FR 1:3: monochromatic red and far red LEDs at ratio 1:3. LEDs used for R:FR lighting treatments were FD-39R-Y 3W 740 and FD-33R-Y 3W 660 nm (Shenzhen Fedy Technology Co., China).

LEDs used for R:FR lighting treatments were FD-39R-Y 3W 740 and FD-33R-Y 3W 660 nm (Shenzhen Fedy Technology Co., Ltd., Guanlan, Shenzhen, China). The light emission spectra of the light sources were determined by an integrating sphere (1 cm diameter opening) coupled with a spectroradiometer OL770 (Optronic Lab. Inc., Orlando, FL, United States) (Figure 1). In all photoperiodic lighting treatments, Photosynthetic Photon Flux Density (PPFD) at canopy level was 3–4 μmol m^–2^ s^–1^. The estimated PPE for each lighting treatment was calculated according to Sager et al. (1988).

### Sampling

Before planting, three tuberous roots per hybrid were sampled. During cultivation, the tuberous root and three fully expanded leaves per plant, per 3 plants per combination *Hybrid* × x *Lighting treatment*, were sampled in the morning (9:00–11:00), during the 7th week after planting (vegetative phase), and the 15th week after planting (flowering phase). After collection, all samples were immediately frozen in liquid nitrogen prior to storage at −80°C, and before the analysis, they were frozen dried at −50°C for 3 days and powdered in a cooled mortar.

### Leaf Photosynthetic Pigments

Photosynthetic pigments were extracted from fully expanded leaves of plants at the 12th week after planting (phase of flowering), on 1 leaf per plants, in 4 plants per combination *Hybrid* × x *Lighting treatment*. Samples of 10 mg lyophilized leaf tissues were homogenized in 1 ml methanol according to Annunziata et al. (2013). The resulting extracts were centrifuged at 4,800*g* for 15 min, and chlorophylls (Chls) *a* and *b* and total carotenoids were estimated by measuring the absorbance of the supernatants at 470, 652, and 665 nm in polypropylene microplates by a microplate reader (Synergy HT; BioTEK Instruments, Bad Friedrichshall, Germany) according to Woodrow et al. (2017).

### Starch, Soluble Sugars, Soluble Proteins, Amino Acids, and Polyphenols in Leaves and Tuberous Roots

Starch and soluble sugars were extracted according to Ferchichi et al. (2018) with some modifications. Frozen powdered tuberous roots and leaves (50 mg) were submitted to a first extraction in 300 ml of ethanol (98%, v/v) at 80 °C for 20 min and centrifuged at 14,000 × *g* at 4°C for 10 min. The clear supernatants were collected in 1 ml tubes and stored at 4°C. The remaining pellets were submitted to two subsequent extractions with 150 ml of ethanol 80% (v/v) and 50% (v/v), respectively, at 80°C for 20 min. The tubes were cooled in ice and centrifuged at 14,000 × *g*, for 10 min at 4°C. The clear supernatants of the two following extractions were pooled together with that of first extraction and stored at −20°C until analysis. The pellets of the ethanol extraction were further used for starch determination, by adding to them 250 ml of 0.1 M KOH and heating at 90°C for 2 h. After cooling in ice, the samples were acidified to pH 4.5 by adding 70 ml of 1 M acetic acid. Aliquots of 100 μl of acidified samples, mixed with 100 μl of an enzymatic hydrolysis buffer constituted by sodium acetate 50 mM pH 4.8, α-amylase 2 U/ml, and amyloglucosidase 20 U/ml, were incubated at 37°C for 18 h. The samples were vortexed and then centrifuged at 13,000 × *g* for 10 min at 4°C, and the supernatant containing the glucose derived from hydrolyzed starch was used for measurement. The enzymatic assay of soluble sugars of the ethanolic extracts as well as glucose originating from starch hydrolysis was performed by a FLX-Xenius spectrophotometer (SAFAS, Monaco) according to Carillo et al. (2019c). Soluble proteins were extracted by mixing 20 mg of lyophilized plant material with 0.1 M Tris–HCl 200 mM pH 7.5 containing 500 mM MgCl_2_ at 4°C for 24 h, the samples were vortexed and then centrifuged at 16,000 × *g*, for 10 min at 4°C, and the clear supernatants were separated from the pellets. Triplicate aliquots of 20 μl of extracts, as well as protein standards prepared according to Carillo et al. (2019b), were dispensed into wells of a polypropylene microplate. The wells contained also 180 μl of Bio-Rad protein assay dye reagent diluted 1:5 with bidistilled water (Bradford, 1976). The solutions were mixed, and then absorbance at 595 nm was recorded on a microplate reader (Synergy HT, BioTEK Instruments, Bad Friedrichshall, Germany). The absorbance of samples was referred to the calibration curve of protein standards used, and the concentration was calculated accounting for dilution factor.

Amino acids were extracted from frozen powdered tuberous roots and leaves by mixing 40 mg samples with 1 ml of ethanol:water in the ratio 40:60 (v/v), incubating overnight at 4°C, and centrifuging at 14, 000 × *g* for 10 min at 4°C (Carillo et al. (2012). The supernatants were pooled and used for the analyses. The primary amino acids were determined by high-performance liquid chromatography (HPLC) according to Ferchichi et al. (2018) using a Shimadzu Nexera X2 UHPLC system (Shimadzu, Italy), after pre-column derivatization of 20 μl of ethanolic extract with 40 μl of o-phthaldialdehyde (OPA) reagent in the an autosampler needle. OPA reagent was prepared by mixing 50 μl of OPA dissolved in methanol (142 mg ml^–1^) and 37 μl of pure β-mercaptoethanol with 1 ml of 0.8 mM Na-borate buffer, pH 10.4. The derivatized sample was then injected onto the column (ZORBAX Eclipse Plus C18, 250 × 4.6 mm internal diameter; Agilent Technologies Italia S.p.A) and eluted at a flow rate of 1 ml min^–1^ at 25°C with a discontinuous gradient as detailed in Carillo et al. (2019c). The amino acid–OPA derivatives were detected by their fluorescence with an excitation at 330 nm and an emission at 450 nm. The HPLC peaks were identified and quantified by comparing their retention time and area data with those obtained from the standards (Carillo et al., 2019c). Proline was determined from the same ethanolic extract by an acid ninhydrin method according to Woodrow et al. (2017). The amino acids were expressed as μmol g^–1^ FW.

The total polyphenols content was determined by the Folin–Ciocalteu method according to Singleton et al. (1999) with few modifications. Lyophilized tissues (30 mg) were extracted in 700 μl of 60% methanol (v:v); 35 μl of extract was mixed with 125 μl of the Folin–Ciocalteu reagent diluted with distilled water (1:4 v/v), and, after mixing for 6 min, 650 μl of 3% (w:v) sodium carbonate was added. After 90 min at room 25°C temperature, the absorbance at 760 nm was determined in a microplate reader (Synergy HT, BioTEK Instruments, Bad Friedrichshall, Germany). The polyphenols concentration was expressed as gallic acid equivalents (GAE) as described by Carillo et al. (2019c).

### Statistical Analysis

The experiment was conducted on 25 plants per combination *Hybrid* × x *Lighting treatment*. Data were analyzed by ANOVA using the SPSS 25 software package^2^, and means were compared by Duncan *post-hoc* test at *p* ≤ 0.05. Three different plants were used for each metabolite analysis, and three plants were used for the analysis of plant growth and flowering.

For all the analyzed parameters, principal component analysis (PCA) was conducted on a number of replicates mentioned above, using the Minitab 18.1 statistical software, aimed to extract trends when multiple qualitative variables were used, by formulating new variables correlated with the original ones (Ciarmiello et al., 2015). The PCA outputs included treatment component scores as well as variable loadings (Ferchichi et al., 2018).

The heat map results used in the pathway maps summarizing the effect of light treatments and hybrids on starch, soluble sugars, free amino acids, including branched-chain amino acids (BCAAs) and methyl ethanolamine (MEA), and polyphenols, were calculated as Logarithm base 2 (Log_2_) of the value to average (avg.) ratio, and visualized using a false-color scale, with red indicating an increase and blue a decrease of values (according to Carillo et al., 2020).

## Results

### Leaf Photosynthetic Pigments

**FIGURE 2:**
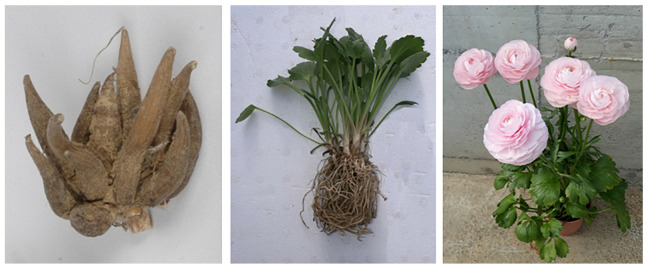
Plant phenological phases in *Ranunculus asiaticus* L.: pre-planting phase (rehydrated and vernalized tuberous roots), vegetative phase, and flowering phase.

At the stage of flowering (Figure 2), the contents of Chl a, Chl b, and carotenoids in MBO leaves were on average 0.05, 0.02, and 0.02 mg g^–1^ DW, respectively, independently of the light treatments (Supplementary Table S2), and they significantly differed (*p* > 0.05) from MBO at the rosette stage (Supplementary Table S1), and from MDR in both the rosette and flowering stages (Supplementary Tables S3, S4). In particular, MDR showed values of Chl a, Chl b, and carotenoids that did not differ significantly between the rosette and flowering stages and were, on average, 0.12, 0.04, and 0.04 mg g^–1^ DW, respectively.

### Starch and Soluble Sugars in Tuberous Roots and Leaves

At the pre-planting stage (Figure 2), pre-sprouted tuberous roots of MBO and MDR hybrids showed similar starch and soluble sugar contents. In particular, the average values for starch, glucose, fructose, and sucrose were 17.2, 25.0, 29.2, and 12.6 mg g^–1^ DW, respectively (Supplementary Tables S1, S4). However, later, the starch content decreased differently in the two hybrids, varying also in dependence on the light conditions. In fact, at the rosette stage, the MBO and MDR tuberous roots had an average starch contents of 11.3 and 9.8 mg g^–1^ DW, respectively. In particular, in MBO-NL was present the highest starch content was present in MBO-NL (13.0 mg g^–1^ DW) (Supplementary Table S1), whereas the lowest starch value of starch was present in MDR-R:FR 1:3 (7.4 mg g^–1^ DW) (Supplementary Table S4). The hexose contents in the tuberous roots of the two hybrids at the rosette stage were similar, but while whereas MBO showed a higher content of glucose, MDR had a higher content of fructose. However, the sucrose content was higher in MDR than in MBO, and in particular, it they were on average 6.0 and 4.3 mg g^–1^ DW, respectively, with the lowest value present in the MDR-R:FR 1:3 (4.0 mg g^–1^ DW). The leaves at the same stage of growth, showed an average content of starch of 16.3 and 13.4 in MBO and MDR, respectively, with the highest and lowest contents of 18.7 and 11.0 mg g^–1^ DW present in MBO-FL and MDR-NL, respectively. However, in the leaves at the rosette stage, the glucose content strongly increased in both hybrids, showing values of 42.4 and 38.7 mg g^–1^ DW in MBO and MDR, respectively (Supplementary Tables S1, S4).

At the flowering stage, while the starch content was stable in the leaves (e.g., 19.0 and 14.0 mg g^–1^ DW on average in MBO and MDR, respectively), it strongly increased in tuberous roots, getting average values of 29.1 and 31.3 mg g^–1^ DW in MBO and MDR hybrids, respectively. Glucose decreased to values of about 32.4 μmol g^–1^ DW in the leaves of both hybrids, whereas sucrose increased in both tuberous roots (Supplementary Tables S2, S5).

### Proteins and Polyphenols in Tuberous Roots and Leaves

At the pre-planting stage, pre-sprouted tuberous roots of MBO and MDR plants showed protein values of 58.2 and 41.4 mg g^–1^ DW, respectively. The germination caused a strong decrease of proteins, which were on average 21.5 mg g^–1^ DW in tuberous root and leaves of MBO plants (Supplementary Table S1), and 21.3 and 28.4 in tuberous root and leaves of MDR plants, respectively (Supplementary Table S4). During the flowering stage, the protein content significantly increased in tuberous roots (+56%, on average), whereas it decreased in leaves (−18%) of MBO hybrids (Supplementary Table S2) compared with the rosette stage. The highest and lowest values of proteins were present in tuberous roots and leaves, respectively, of both FL and R:FR 3:1 lighting treatments. Protein contents significantly (*p* > 0.05) varied also among lighting treatments in both in tuberous roots and leaves (Supplementary Table S3). In MDR plants under the flowering stage, the proteins in leaves did not show any significant change compared with the previous phenological stage, but they significantly differed among lighting treatments (Supplementary Table S6). Instead, in tuberous roots under the flowering stage, the proteins significantly increased (+40%) compared with the rosette stage, but their content was independent of lighting treatments (Supplementary Tables S2, S3).

During the pre-germination stage, polyphenol content contents were 6.0 and 5.4 μg g^–1^ DW in MBO and MDR tuberous roots, respectively (Supplementary Tables S1, S4). In the successive rosette stage, the content of this metabolite slightly varied in tuberous roots of MBO plants, whereas it increased to a value of 10.3, on average, in leaves, which was independent of lighting treatments and the phenological stage (Supplementary Tables S1, S2). During the flowering stage, polyphenols in MBO tuberous roots significantly increased compared with those present in the rosette stage, except for FL lighting treatment, which remained stable (Supplementary Tables S2, S3). In the MDR hybrid, during the rosette stage, the content of polyphenols underwent the same increase in leaves and tuberous roots up to 10.25 μg g^–1^ DW, on average, except for the R:FR 1:3 lighting treatment (Supplementary Table S4). However, in MDR plants during the flowering stage, the content of polyphenols in tuberous roots significantly (*p* < 0.05) changed in NL (+43%) and FL (−27%) lighting treatments, whereas it remained stable in the other ones. In the MDR leaves slightly but significantly changed, but their average value remained stable (Supplementary Tables S5, S6).

### Free Amino Acids in Tuberous Roots and Leaves

**FIGURE 3:**
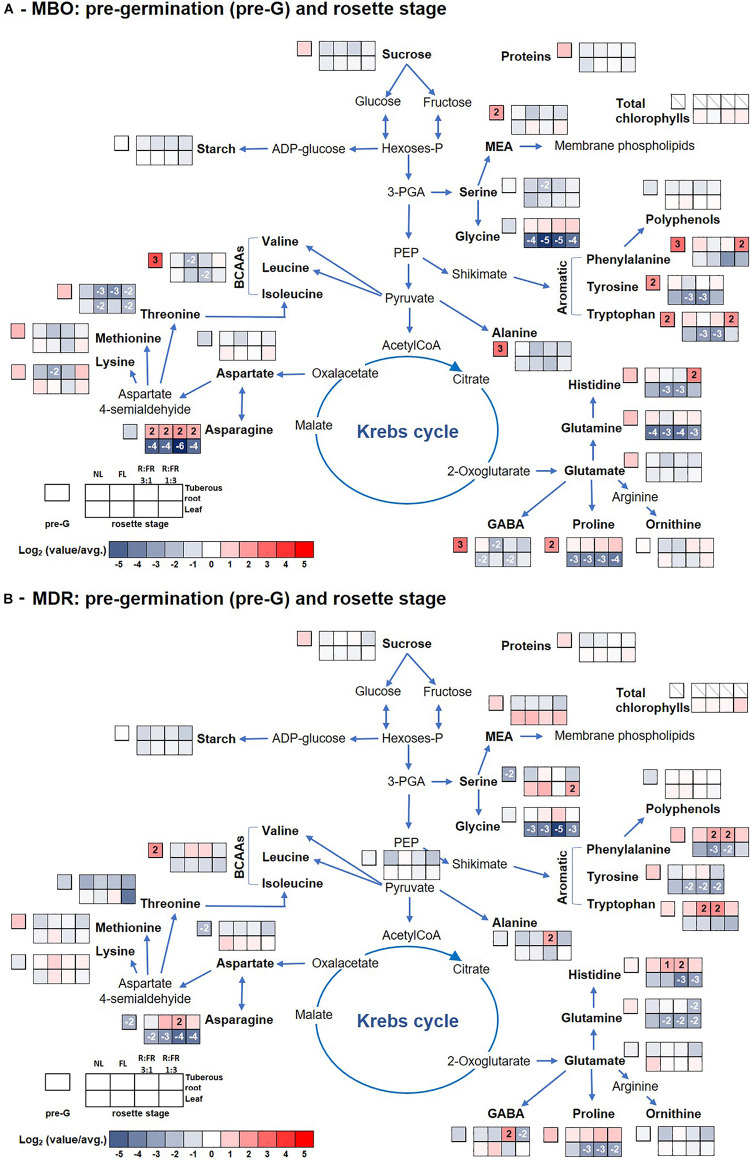
Pathway map summarizing the effect of growth stage (pre-germination and leaf rosette) and photoperiodic lighting treatments: NL, NL + photoperiodic lighting with fluorescence light (FL), NL + photoperiodic lighting with LEDs R:FR 3:1 (R:FR 3:1), and NL + photoperiodic lighting with LEDs R:FR 1:3 (R:FR 1:3), on leaves and tuberous roots of the two hybrids MBO **(A)** and MDR **(B)** of *Ranunculus asiaticus* L. The heat map results were calculated as Logarithm base 1.5 (Log_1.5_) of single values/total average values and visualized using a false-color scale, with red indicating an increase and blue a decrease. The significant ratios were indicated in the squares.

**FIGURE 4:**
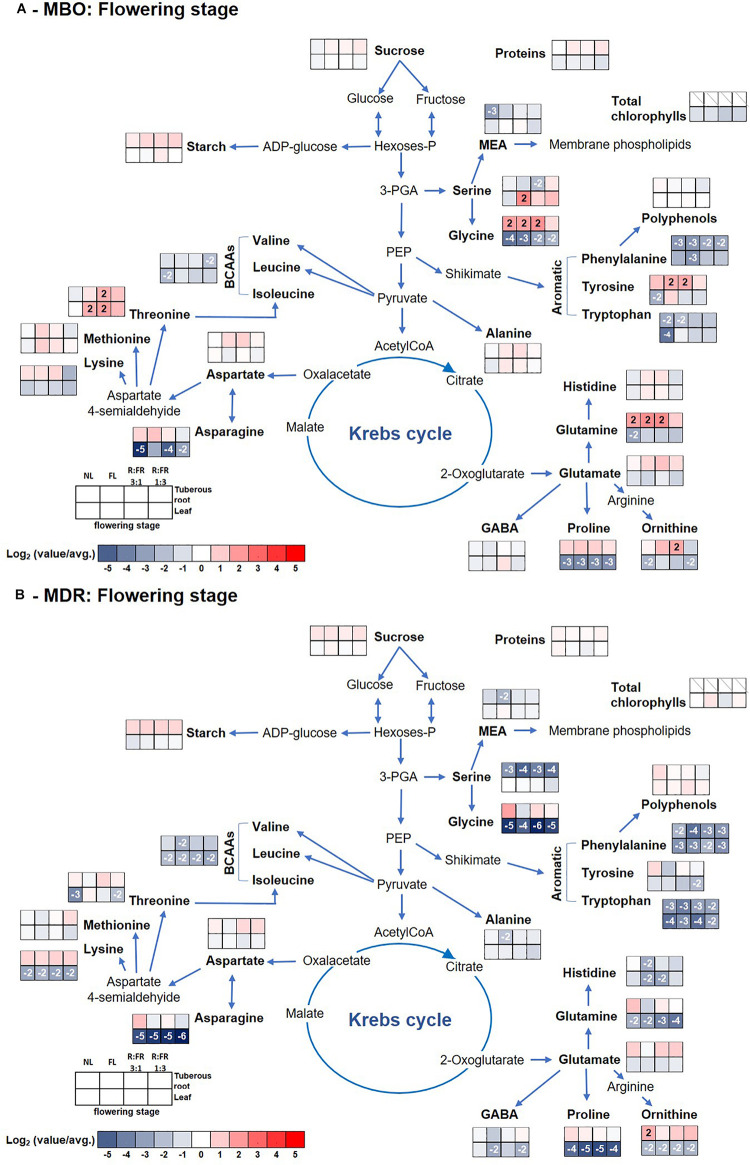
Pathway map summarizing the effect of growth stage (flowering) and photoperiodic lighting treatments: NL, NL + photoperiodic lighting with fluorescence light (FL), NL + photoperiodic lighting with LEDs R:FR 3:1 (R:FR 3:1), and NL + photoperiodic lighting with LEDs R:FR 1:3 (R:FR 1:3), on leaves and tuberous roots of the two hybrids MBO **(A)** and MDR **(B)** of *Ranunculus asiaticus* L. The heat map results were calculated as Logarithm base 1.5 (Log_1.5_) of single values/total average values and visualized using a false-color scale, with red indicating an increase and blue a decrease. The significant ratios were indicated in the squares.

The content of total amino acids was higher in MBO than in MDR hybrid independently of the growth stage and light conditions, except for those present in leaves of MDR plants during the rosette stage independently of lighting treatments (Supplementary Tables S1, S2, S4, S5). The main free amino acids in tuberous roots during the pre-planting stage were glutamine (190 and 117 μmol g^–1^ DW in MBO and MDR, respectively), minor amino acids, as the sum of histidine, isoleucine, leucine, lysine, methionine, phenylalanine, tryptophan, tyrosine, and valine (152 and 62 μmol g^–1^ DW in MBO and MDR, respectively), and branched chain amino acids (BCAAs), as the sum of isoleucine, leucine and valine (79 and 32 μmol g^–1^ DW in MBO and MDR, respectively), followed by glutamate, threonine, asparagine, and alanine (Supplementary Tables S1, S4 and Figures 3A,B). γ-*Aminobutyric acid* (GABA), alanine, threonine, serine, glutamate, minor amino acids, and total amino acids were 16.3, 9.8, 4.9, 4.2, 2.6, 2.4, and 2.3- fold higher in MBO than in MDR tuberous roots during the pre-planting stage, respectively. During the following growth stages, there was a remodulation of amino acid contents in the plant tissues, however, they remained always higher in tuberous roots than in leaves, independently of the growth stage. In order to obtain an easier overview of the changes of amino acids profile, pathway maps were constructed choosing for the heat maps to make the ratio of single parameter values to the total average values over the hybrid, growth stage, and light treatments to evidence not only the single changes but also the relative ones. The pathway maps were also included carbohydrates, proteins, and polyphenols (Figures 3,4).

Glutamate and threonine were the only amino acids that strongly increased in leaves of the MDR rosette and MBO flowering stages, respectively, independently of light treatments, peaking the former at NL (39 μmol g^–1^ DW) (Supplementary Table S4 and Figure 3B) and the latter at R:FR 1:3 light treatments (81 μmol g^–1^ DW) (Supplementary Table S2 and Figure 4A). On the contrary in tuberous roots, asparagine strongly increased during the rosette stage, and in particular in MBO plants (+722%), independently of light treatments, peaking at R:FR 3:1 with a content of 379 μmol g^–1^ DW (Supplementary Tables S1, S4 and Figures 3A,B). This amino acid remained at high levels in the same tissues in both hybrids, also during the flowering stage, even if it varied among light treatments (Supplementary Table S2, S5 and Figures 4A,B). The glycine content of tuberous roots was always higher in the rosette and flowering stage stages than in the pre-germination stage. In particular, it strongly increased in MBO at the flowering stage in all light conditions (+507%) except for R:FR 1:3. Glutamine was the second more abundant amino acid in tuberous roots during the rosette and flowering stages. However, it peaked in all treatments of MBO plants during the flowering stage except for the MBO-R:FR 1:3 treatment, as glycine (Supplementary Table S2 and Figure 4A).

### Principal Component Analysis of the Analyzed Parameters

**FIGURE 5:**
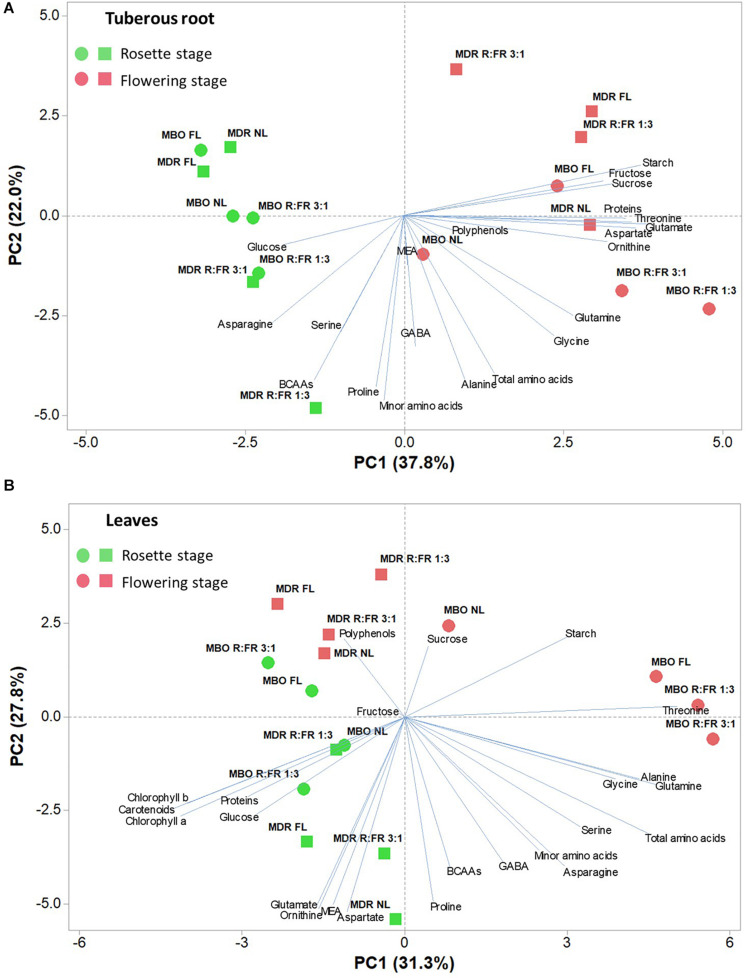
Principal component loading plot and scores of principal component analysis (PCA) of starch, soluble carbohydrates, photosynthetic pigments (only for leaves), polyphenols, soluble proteins, and amino acids of tuberous roots **(A)** and leaves **(B)** of two hybrids (MBO and MDR) of *Ranunculus asiaticus* L. at the growth stage of rosette and flowering. The plants underwent different photoperiodic lighting treatments: NL, NL + photoperiodic lighting with fluorescence light (FL), NL + photoperiodic lighting with LEDs R:FR 3:1 (R:FR 3:1), and NL + photoperiodic lighting with LEDs R:FR 1:3 (R:FR 1:3).

To obtain an in-depth overview of the metabolites profile of the two hybrids under the different lighting treatments and growth stages, a PCA was conducted for all of the above-mentioned measured parameters for both tuberous roots and leaves (Figures 5A,B).

The PCA of all analyzed parameters highlighted that the first three principal components (PCs) were related with eigenvalues higher than 1 and explained 72% of the total variance for tuberous roots, with PC1, PC2, and PC3 accounting for 37.8, 22.0, and 12.1%, respectively, and 74.2% of the total variance for leaves, with PC1, PC2, and PC3 accounting for 31.3, 27.8, and 15.1%, respectively (data not shown). The growth stage contributed to the clear separation on PC1 for tuberous roots and on PC2 for leaves (Figure 5). In tuberous roots, PC1 was positively correlated with glutamate, starch, aspartate, threonine, proteins, sucrose, ornithine, and fructose. PC1 was also negatively correlated with asparagine, glucose, BCAAs, and serine. PC2 was negatively correlated with minor amino acids, proline, BCAAs, alanine, total amino acids, GABA, glycine, and serine (Figure 5A). In leaves, PC1 was positively correlated with glutamine, total amino acids, alanine, glycine, serine, starch, and asparagine, whereas it was negatively correlated with carotenoids, Chl a, Chl b, proteins, and glucose. PC2 was positively correlated with starch, polyphenols, and sucrose, whereas it was negatively correlated with MEA, ornithine, glutamate, aspartate, and proline (Figure 5B). In addition, the MBO-R:FR 3:1 treatment showed the highest minor amino acids content, in particular BCAAs, whereas the MDR-R:FR 3:1 treatment had the highest photosynthetic pigments and polyphenols content (Figure 5).

## Discussion

MBO and MDR tuberous roots at the pre-germination stage, after vernalization, showed similar contents of starch, soluble carbohydrates, and polyphenols, but a completely different profile of amino acids. In particular, GABA and alanine strongly increased in MBO tuberous roots compared with MDR ones. Yoshida et al. (1999) demonstrated that cold exposure induces the inactivation of cell H^+^-ATPases and the impairment of the H^+^ extrusion from cytosol, with the consequent rapid acidification of the cytoplasm and the concomitant alkalization of the vacuoles. In this view, the increases of alanine, deriving from the malic enzyme decarboxylation of malate to pyruvate, and GABA, deriving from the decarboxylation of glutamate catalyzed by glutamate decarboxylase, are proton-consuming reactions able to buffer cytoplasmic acidosis and regulate pH (Carillo, 2018; Carillo et al., 2019c; Van Oosten et al., 2019). Subsequently, after the relief from cold stress, the two amino acids can be converted to intermediates of the TCA cycle and used to produce alpha-keto acids and ATP (Bouché and Fromm, 2004). In addition, GABA exerts a well-known ROS scavenger activity able to stabilize and protect membranes and macromolecules from oxidative stress (Molina-Rueda et al., 2015).

At the germination stage, MBO tuberous roots under NL treatment still retained high contents of starch and glutamine and accumulated MEA, an amino acid derivative important for the synthesis and/or regeneration of phospholipids, derived by the decarboxylation of serine. Instead, all the other MBO tuberous roots exposed to the other light conditions were able to remobilize the N reserves synthetizing the amides asparagine and/or glutamine, amino acids having the highest nitrogen:carbon ratios and used for long-distance transport of nitrogen throughout the plant (Lea et al., 2007).

The increase of polyphenols in MDR tuberous roots during the germination stage evidenced a delayed response to the oxidative stress caused by vernalization (Yanagida et al., 2004). The increase of these metabolites was accompanied by an enhancement of the content of glucose under NL, proline under FL, fructose, sucrose, minor amino acids, and in particular BCAAs under R:FR 3:1, and by high amounts of GABA and alanine in addition to soluble carbohydrates and amino acids under R:FR 1:3 treatments. Both soluble carbohydrates and amino acids can play a role in protecting membranes and macromolecules by ROS (Woodrow et al., 2017). In particular, the photoperiodic treatment R:FR 3:1 and R:FR 1:3 determined an increase of minor amino acids including BCAAs, which may actively function as antioxidants and alternative electron donors for the mitochondrial electron transport chain (Woodrow et al., 2017), limiting the occurrence of oxidative damages. Their increase appeared to be related to the levels of fructose, in agreement with Fritz et al. (2006) and Carillo et al. (2019a). Moreover, fructose, or glucose itself, can also be used for supplying glucose to the oxidative pentose pathway (OPP) and enhancing NADPH production, which is a major cofactor of the ROS scavenging ascorbate–glutathione pathway (Couée et al., 2006). However, the synthesis and accumulation of these metabolites with a protective function has a very high cost in terms of energy consumption (50–70 mol ATP for mole) (Raven, 1985; Cirillo et al., 2019) and seems to require the intervention of recent photosynthates from leaves. This export of carbohydrates from leaves at the germination stage, necessary for repairing the damages caused by vernalization and not promptly repaired at the pre-germination stage, could be responsible for the delay in growth and flowering in MDR plants.

At the flowering stage, in MBO under R:FR 1:3 light treatment, there was an increase of starch and amino acids, in particular GABA, otherwise Chls, carotenoids, polyphenols, and proteins decreased, while as reported by Modarelli et al. (2020b), leaf area still enlarged. Accordingly, Heraut-Bron et al. (2011) reported that white clover leaves that developed under a low R:FR ratio showed a decrease of photosynthetic pigments without consequences on the net photosynthesis, while leaf area increased. In these conditions, it is probable that the strong increase of GABA could be related to the decrease of protection of the photosynthesis apparatus deriving from the decrease of carotenoids and polyphenols. Both classes of metabolites are well known for their important ROS scavenging activity (Sandmann, 2019). Carotenoids, in particular, avoid photoinhibition under rapid fluctuations in light intensity when photochemical quenching activity is exceeded (Külheim et al., 2002). In addition, carotenoids protect chloroplasts from excess light, modulating the non-radiative dissipation of excess excitation energy and mediating the direct quenching of chlorophyll triplets (3Chl^∗^) (Cazzaniga et al., 2012). The increase of starch proved that the photosynthesis was working quite efficiently and well protected by GABA, but this latter was not used for investing proteins in new leaves (Modarelli et al., 2020b). Low R:FR is, in fact, perceived by plants as a signal of shade conditions and activates a set of genes related to cell proliferation and/or enlargement among which while decreasing cytokinin in young, middle, and old leaves (Wu et al., 2017). Auxin is well known for its induction of acid growth, in which plant cells and plant cell walls elongate and/or expand quickly without effectively growing (Arsuffi and Braybrook, 2017). Indeed, it is known that the addition of FR to the natural light spectrum (reducing the R:FR ratio and the PPE value at plant level) promotes leaf expansion (Li and Kubota, 2009; Park and Runkle, 2017,2018; Fukuda, 2019; Meng and Runkle, 2019). These changes in metabolism allow plants to enact a ubiquitous mechanism known as “shade-avoidance syndrome” in which leaves already present are enlarge, and flowering is accelerated (Martínez-García et al., 2014; Modarelli et al., 2020b). In these conditions, large amounts of amino acids are exported to roots but not sugars.

MBO R:FR 3:1 underwent a slower decrease of photosynthetic pigments in leaves, but an equally fast export of amino acids to tuberous roots that were also converted to proteins. MBO NL showed a fast decrease of leaf metabolites, but not an equally and quick filling of the tuberous root reserve. Unexpectedly, FL, the other high R:FR condition, was faster and more efficient than the other MBO lighting conditions to transport sugars and amino acids to tuberous roots and by converting them to starch, proteins, and serine.

Except for MDR R:FR 1:3, that accelerated the export of metabolites from leaves without efficiently replenishing the sugar reserves of tuberous roots, all the other lighting conditions showed the presence of a still active photosynthetic apparatus, as proven by the persistence of photosynthetic pigments and proteins, but an already active export of sugars, soon converted to starch. MDR NL showed the lowest starch content in tuberous roots at the flowering stage but the highest content of glucose and polyphenols.

The study of metabolic profiling confirmed the intrinsic differences between the two *R. asiaticus* hybrids. The different profiles of tuberous root reserves seem responsible for the capacity of MBO to exert a faster overcome of the vernalization stress and an equally fast development of the germination and flowering activities. Some changes in morphological and physiological traits were certainly related to the different responses to day length and photoperiodic light quality in the two hybrids, as also observed in plants from rehydrated and vernalized roots (Modarelli et al., 2020a).

Photoperiodic treatments with all the lighting sources were effective to reduce the flowering time in both the hybrids, even though R:FR light at 1:3 ratio was less efficient than the other treatments in MDR. The metabolic pathway and PCA carried out in this work facilitated a broad view of morpho-physiological and biochemical traits and enabled the identification of phenotypic variation patterns associated with *Ranunculus* genotype and/or lighting conditions, as evidenced also for other species (Carillo et al., 2019a,c).

In conclusion, in its entirety, our work provides information on the different performances of two *R. asiaticus* L. hybrids in a cold glasshouse in a Mediterranean environment and on their response to photoperiodic treatment, in terms of duration and light spectrum. The two hybrids confirmed intrinsic differences in metabolic profile, as already observed in photosynthetic behavior, plant growth, and flowering (Carillo et al., 2019a; Modarelli et al., 2020b,c).

Our results demonstrate that, together with the different behaviors depending on the plant genotype, the different sensitivities of the hybrids to vernalization should be considered for the proper choice of the preparation procedure in production scheduling at large scale. Similarly, the specific response of the hybrids to light environment should be taken into account to optimize lighting protocols and production planning in *R. asiaticus* L.

## Data Availability Statement

The raw data supporting the conclusions of this article will be made available by the authors, without undue reservation.

## Author Contributions

RP and SD designed the experiment. GM carried out the plant cultivation and performed the statistical analysis of data. ED’A and GF performed the pigments and metabolic analyses. PC performed the PCA and constructed the metabolic pathways. RP and PC wrote the manuscript. All authors contributed to the article and approved the submitted version.

## Conflict of Interest

The authors declare that the research was conducted in the absence of any commercial or financial relationships that could be construed as a potential conflict of interest.

## Footnotes

1 https://www.bianchericreazioni.it/

2 www.ibm.com/software/analytics/spss

## Abbreviations

ABA: abscisic acid
BCAAs: branched-chain amino acids
Chl: chlorophyll
DW: dry weight
EOD: end-of-day
F0: basal fluorescence
FL: fluorescence lamp
Fm: maximal fluorescence in the dark
FR: Far Red light
Fv: difference between the maximal and the basal fluorescence
Fv/Fm: maximal PSII photochemical efficiency
GA: gibberellic acid
*GABA*, γ -*aminobutyric acid;* GAE: gallic acid equivalents
LD: long day
LEDs: light emitting diodes
MEA: methyl ethanolamine
NL: natural day length
PCs: principal components
Pfr: phytochrome far red
PPE: phytochrome photoequilibria
PPFD: Photosynthetic Photon Flux Density
Pr: phytochrome red
Ptot: total amount of phytochrome
R: red light
ROS: reactive oxygen species.
