# Supplementary material for: Metabolic Profile and Performance Responses of Ranunculus asiaticus L. Hybrids as Affected by Light Quality of Photoperiodic Lighting
Source: Front Plant Sci. 2020 Nov 19;11:597823. doi: 10.3389/fpls.2020.597823 (PMC7727310; doi:10.3389/fpls.2020.597823)
Supplement: Supplementary file 1 [file Data_Sheet_1.docx]

**Supplemental table 1**: Chlorophyll *a*, *b*, carotenoids, polyphenols (in µg g^-1^ DW), soluble proteins, starch, glucose, fructose, sucrose (in mg g^-1^ DW), and free amino acid content (in µmol g^-1^ DW) in tuberous roots and leaves of *Ranunculus asiaticus* L. plants hybrid MBO at pre-germination and leaf rosette stage; growth after pre-germination in a cold greenhouse under four photoperiodic lighting treatments: NL, NL + photoperiodic lighting with fluorescence light (FL), NL + photoperiodic lighting with LEDs R:FR 3:1 (R:FR 3:1), NL + photoperiodic lighting with LEDs R:FR 1:3 (R:FR 1:3). Mean values ± SE; n = 3.

|  | MBO | | | | | | | | | | | | | | | |
| --- | --- | --- | --- | --- | --- | --- | --- | --- | --- | --- | --- | --- | --- | --- | --- | --- |
|  |  | NL | | FL | | | R:FR 3:1 | | | R:FR 1:3 | | |  | |  | |
| Phenological stage | Pre-Germination | Leaf rosette | | | | | | | | | | | | | | |
| Plant organ | Tuberous roots | Tuberous roots | Leaves | Tuberous roots | Leaves | Tuberous roots | | Leaves | Tuberous roots | | Leaves | *Mean (Tuberous roots)* | | *Mean (Leaves)* | |  |
| Chl *a* |  |  | 0.13±0.01 |  | 0.09±0.01 |  | | 0.13±0.01 |  | | 0.14±0.01 |  | | *0.12* | |  |
| Chl *b* |  |  | 0.03±0.00 |  | 0.03±0.00 |  | | 0.04±0.00 |  | | 0.04±0.01 |  | | *0.03* | |  |
| Carotenoids |  |  | 0.04±0.00 |  | 0.03±0.00 |  | | 0.03±0.00 |  | | 0.04±0.00 |  | | *0.04* | |  |
| Soluble proteins | 58.2±1.4 | 18.5±0.9 | 15.2±1.0 | 20.1±0.8 | 28.1±0.4 | 23.0±0.1 | | 24.6±0.7 | 22.3±1.6 | | 20.3±1.5 | *21.0* | | *22.1* | |  |
| Starch | 17.0±0.6 | 13.0±2.4 | 17.5±0.9 | 10.7±0.8 | 18.7±3.7 | 10.8±0.7 | | 16.4±0.4 | 10.9±0.5 | | 12.3±0.3 | *11.3* | | *16.2* | |  |
| Glucose | 26.6±2.5 | 26.9±0.4 | 39.5±0.8 | 28.8±0.7 | 44.9±0.4 | 19.6±0.4 | | 40.1±0.5 | 23.8±1.0 | | 45.1±1.2 | *24.8* | | *42.4* | |  |
| Fructose | 28.8±0.6 | 9.85±0.56 | 21.9±0.5 | 11.5±0.4 | 22.8±0.7 | 8.82±0.28 | | 25.3±0.2 | 13.6±0.4 | | 24.2±0.5 | *10.9* | | *23.6* | |  |
| Sucrose | 12.5±0.2 | 3.88±0.25 | 5.07±0.16 | 4.54±0.18 | 5.86±0.22 | 3.37±0.10 | | 6.18±0.05 | 5.45±0.34 | | 5.95±0.22 | *4.31* | | *5.76* | |  |
| Polyphenols | 6.00±0.15 | 6.82±0.10 | 10.1±0.4 | 7.34±0.10 | 11.3±0.2 | 6.10±0.18 | | 9.65±0.27 | 7.64±0.13 | | 10.2±0.5 | *6.98* | | *10.3* | |  |
| Alanine | 42.0±3.0 | 5.49±0.62 | 3.62±0.22 | 2.07±0.13 | 2.82±0.47 | 3.11±0.26 | | 2.04±0.06 | 3.09±0.25 | | 4.39±0.45 | *3.44* | | *3.22* | |  |
| Arginine | 40.0±2.6 | 2.67±0.32 | 5.49±0.21 | 1.25±0.08 | 3.00±0.37 | 2.19±0.36 | | 2.26±0.06 | 5.28±0.65 | | 5.08±0.53 | *2.85* | | *3.96* | |  |
| Asparagine | 44.0±3.3 | 286±35 | 5.02±0.13 | 311±27 | 6.41±1.07 | 379±39 | | 1.83±0.05 | 294±25 | | 6.22±0.71 | *318* | | *4.87* | |  |
| Aspartate | 2.60±0.04 | 4.26±0.50 | 6.57±0.29 | 2.60±0.27 | 5.63±0.80 | 4.89±0.42 | | 5.06±0.19 | 3.93±0.29 | | 7.04±0.50 | *3.92* | | *6.07* | |  |
| GABA | 34.8±3.5 | 5.53±0.85 | 1.67±0.17 | 1.28±0.09 | 3.17±0.58 | 2.94±0.34 | | 1.67±0.05 | 2.03±0.23 | | 3.33±0.42 | *2.94* | | *2.46* | |  |
| Glutamate | 45.1±3.0 | 18.6±2.3 | 15.5±0.3 | 10.4±0.8 | 18.2±3.0 | 15.4±1.5 | | 14.9±0.4 | 13.7±1.1 | | 20.0±2.1 | *14.5* | | *17.1* | |  |
| Glutamine | 190±14 | 115±14 | 6.57±0.30 | 56.6±4.39 | 7.82±1.16 | 104±11 | | 6.15±0.17 | 108±9.26 | | 11.3±1.24 | *96.2* | | *7.96* | |  |
| Glycine | 4.28±0.37 | 10.1±0.88 | 0.54±0.09 | 11.2±0.7 | 0.20±0.03 | 14.3±1.1 | | 0.29±0.01 | 14.1±1.4 | | 0.60±0.09 | *12.4* | | *0.41* | |  |
| Histidine | 1.49±0.11 | 0.80±0.10 | 0.17±0.02 | 0.58±0.03 | 0.09±0.02 | 0.49±0.05 | | 0.11±0.00 | 3.43±0.20 | | 0.21±0.02 | *1.32* | | *0.15* | |  |
| Isoleucine | 25.0±1.7 | 1.33±0.15 | 1.41±0.02 | 0.46±0.04 | 0.74±0.09 | 0.71±0.06 | | 0.68±0.02 | 1.63±0.14 | | 1.07±0.09 | *1.03* | | *0.97* | |  |
| Leucine | 16.7±1.2 | 0.93±0.10 | 2.87±0.09 | 0.36±0.02 | 1.17±0.15 | 0.53±0.05 | | 0.94±0.03 | 1.28±0.11 | | 2.44±0.23 | *0.78* | | *1.85* | |  |
| Lysine | 3.50±0.38 | 0.63±0.10 | 2.75±0.29 | 0.36±0.01 | 2.01±0.36 | 0.64±0.08 | | 1.09±0.03 | 3.81±0.48 | | 2.52±0.28 | *1.36* | | *2.09* | |  |
| MEA | 13.1±1.1 | 2.95±0.48 | 2.31±0.24 | 1.38±0.14 | 4.32±0.41 | 2.39±0.30 | | 2.90±0.08 | 1.98±0.25 | | 4.47±0.49 | *2.18* | | *3.50* | |  |
| Methionine | 0.51±0.04 | 0.13±0.02 | 0.21±0.01 | 0.07±0.00 | 0.15±0.02 | 0.08±0.01 | | 0.08±0.00 | 0.15±0.01 | | 0.24±0.04 | *0.11* | | *0.17* | |  |
| Ornithine | 1.19±0.02 | 0.61±0.05 | 0.33±0.03 | 0.56±0.04 | 0.30±0.05 | 1.51±0.15 | | 0.30±0.01 | 1.45±0.17 | | 0.23±0.01 | *1.03* | | *0.29* | |  |
| Phenylalanine | 5.84±0.41 | 1.20±0.15 | 1.32±0.06 | 0.61±0.06 | 0.71±0.11 | 0.93±0.09 | | 0.73±0.02 | 4.19±0.36 | | 1.43±0.16 | *1.73* | | *1.05* | |  |
| Proline | 11.4±0.3 | 3.10±0.34 | 7.42±0.32 | 3.49±0.23 | 3.69±0.21 | 4.24±0.08 | | 3.50±0.06 | 5.31±0.08 | | 3.81±0.12 | *4.04* | | *4.60* | |  |
| Serine | 7.57±0.69 | 4.12±0.68 | 3.24±0.35 | 3.03±0.38 | 5.24±1.14 | 4.07±0.58 | | 5.81±0.16 | 6.72±1.03 | | 6.77±0.88 | *4.49* | | *5.27* | |  |
| Threonine | 48.8±4.3 | 9.72±1.51 | 15.4±0.8 | 3.81±0.35 | 5.12±0.76 | 2.70±0.35 | | 11.0±0.3 | 6.91±0.81 | | 6.18±0.76 | *5.77* | | *9.40* | |  |
| Tryptophan | 4.61±0.33 | 1.43±0.18 | 0.31±0.01 | 0.87±0.05 | 0.15±0.03 | 1.43±0.13 | | 0.14±0.00 | 4.22±0.38 | | 0.55±0.08 | *1.99* | | *0.29* | |  |
| Tyrosine | 16.7±1.3 | 4.31±0.51 | 0.90±0.04 | 2.09±0.15 | 0.53±0.08 | 3.60±0.37 | | 0.42±0.01 | 4.41±0.42 | | 1.02±0.13 | *3.60* | | *0.72* | |  |
| Valine | 37.7±2.5 | 3.20±0.37 | 2.12±0.05 | 1.16±0.11 | 1.06±0.14 | 2.41±0.22 | | 0.80±0.02 | 5.19±0.43 | | 1.70±0.15 | *2.99* | | *1.42* | |  |
| BCAAs | 79.5±5.3 | 5.45±0.62 | 6.40±0.06 | 1.98±0.17 | 2.97±0.39 | 3.65±0.33 | | 2.41±0.07 | 8.09±0.68 | | 5.21±0.47 | *4.79* | | *4.25* | |  |
| Minor AA | 152±10 | 16.6±2.0 | 17.6±0.7 | 7.81±0.52 | 9.60±1.19 | 13.1±1.4 | | 7.24±0.19 | 33.6±3.0 | | 16.2±1.7 | *17.8* | | *12.7* | |  |
| Total AA | 596±43 | 483±59 | 85.7±3.2 | 415±34 | 72.5±9.9 | 552±56 | | 62.6±1.7 | 496±42 | | 90.5±9.3 | *486* | | *77.8* | |  |

**Supplemental table 2**: Chlorophyll *a*, *b*, carotenoids, polyphenols (in µg g^-1^ DW), soluble proteins, starch, glucose, fructose, sucrose (in mg g^-1^ DW), and free amino acid content (in µmol g^-1^ DW) in leaves and tuberous roots of *Ranunculus asiaticus* L. plants hybrid MBO at flowering stage; growth after pre-germination in a cold greenhouse under four photoperiodic lighting treatments: NL, NL + photoperiodic lighting with fluorescence light (FL), NL + photoperiodic lighting with LEDs R:FR 3:1 (R:FR 3:1), NL + photoperiodic lighting with LEDs R:FR 1:3 (R:FR 1:3). Mean values ± SE; n = 3.

|  | MBO | | | | | | | | | |
| --- | --- | --- | --- | --- | --- | --- | --- | --- | --- | --- |
|  | NL | | FL | | R:FR 3:1 | | R:FR 1:3 | |  |  |
| Phenological stage | Flowering | | | | | | | |  |  |
| Plant organ | Tuberous roots | Leaves | Tuberous roots | Leaves | Tuberous roots | Leaves | Tuberous roots | Leaves | *Mean (Tuberous roots)* | *Mean (Leaves)* |
| Chl *a* |  | 0.06±0.01 |  | 0.05±0.01 |  | 0.06±0.01 |  | 0.04±0.00 |  | *0.05* |
| Chl *b* |  | 0.02±0.00 |  | 0.02±0.00 |  | 0.02±0.00 |  | 0.01±0.00 |  | *0.02* |
| Carotenoids |  | 0.02±0.00 |  | 0.02±0.00 |  | 0.02±0.00 |  | 0.01±0.00 |  | *0.02* |
| Soluble proteins | 26.7±0.6 | 18.9±0.4 | 37.5±0.9 | 15.6±1.3 | 35.4±0.6 | 19.8±0.7 | 31.1±1.3 | 18.8±0.5 | *32.7* | *18.3* |
| Starch | 23.6±0.5 | 17.9±0.3 | 32.4±1.6 | 18.2±1.7 | 29.8±1.3 | 16.0±0.8 | 30.5±1.0 | 23.6±1.5 | *29.1* | *19.0* |
| Glucose | 28.6±2.4 | 33.4±0.5 | 14.8±0.8 | 33.2±1.0 | 15.7±0.8 | 25.8±0.5 | 19.8±0.5 | 34.7±0.1 | *19.7* | *31.8* |
| Fructose | 14.8±1.7 | 22.2±0.3 | 20.3±1.0 | 23.5±0.6 | 16.9±0.8 | 25.1±0.6 | 16.8±0.5 | 25.9±0.2 | *17.2* | *24.2* |
| Sucrose | 5.70±0.74 | 5.78±0.09 | 9.54±0.56 | 6.14±0.05 | 8.21±0.21 | 6.79±0.19 | 8.21±0.09 | 6.94±0.08 | *7.92* | *6.41* |
| Polyphenols | 10.8±0.1 | 9.97±0.07 | 6.34±0.29 | 9.75±0.25 | 8.60±0.30 | 9.56±0.14 | 8.51±0.17 | 10.2±0.2 | *8.57* | *9.88* |
| Alanine | 5.57±0.61 | 4.14±0.51 | 6.79±0.66 | 5.62±0.60 | 8.32±1.24 | 8.40±0.40 | 9.54±0.67 | 7.50±0.45 | *7.56* | *6.42* |
| Arginine | 5.14±0.60 | 2.10±0.33 | 2.98±0.33 | 3.33±0.35 | 6.34±0.86 | 6.46±0.61 | 6.91±0.55 | 3.37±0.31 | *5.34* | *3.81* |
| Asparagine | 169±15 | 2.14±0.31 | 67.8±8.6 | 17.7±2.1 | 215±26 | 21.0±3.4 | 130±8 | 5.09±0.34 | *146* | *11.5* |
| Aspartate | 5.44±0.51 | 5.66±0.80 | 6.14±0.59 | 4.22±0.39 | 8.53±1.17 | 3.90±0.59 | 9.99±0.73 | 5.55±0.30 | *7.52* | *4.83* |
| GABA | 4.02±1.56 | 3.57±0.59 | 4.03±0.55 | 3.06±0.53 | 3.17±0.28 | 3.31±0.25 | 4.90±0.53 | 7.09±0.80 | *4.03* | *4.26* |
| Glutamate | 25.6±1.9 | 13.5±2.1 | 36.6±4.2 | 10.2±1.1 | 38.9±4.9 | 10.9±1.6 | 51.5±3.3 | 16.2±0.7 | *38.1* | *12.7* |
| Glutamine | 292±24 | 16.6±2.3 | 160±13 | 29.3±3.1 | 384±54 | 37.4±5.5 | 395±25.43 | 33.5±1.5 | *308* | *29.2* |
| Glycine | 29.0±3.1 | 0.61±0.08 | 12.5±1.8 | 2.06±0.13 | 28.3±2.1 | 0.70±0.06 | 35.1±2.7 | 1.57±0.05 | *26.2* | *1.24* |
| Histidine | 0.46±0.05 | 0.48±0.07 | 0.37±0.05 | 0.46±0.04 | 0.92±0.09 | 0.87±0.11 | 0.98±0.06 | 0.79±0.06 | *0.68* | *0.65* |
| Isoleucine | 0.75±0.12 | 0.36±0.06 | 0.24±0.02 | 0.66±0.06 | 0.53±0.05 | 1.21±0.37 | 0.54±0.08 | 0.46±0.02 | *0.52* | *0.67* |
| Leucine | 0.74±0.11 | 0.64±0.09 | 0.28±0.03 | 0.90±0.08 | 0.58±0.08 | 1.13±0.15 | 0.68±0.08 | 0.95±0.05 | *0.57* | *0.90* |
| Lysine | 2.68±0.38 | 0.74±0.07 | 0.46±0.05 | 0.60±0.06 | 2.70±0.06 | 0.98±0.07 | 3.13±0.39 | 0.87±0.08 | *2.24* | *0.80* |
| MEA | 0.59±0.11 | 2.27±0.55 | 2.38±0.38 | 1.87±0.28 | 1.61±0.21 | 3.60±0.27 | 2.57±0.34 | 3.80±0.29 | *1.79* | *2.88* |
| Methionine | 0.18±0.01 | 0.15±0.02 | 0.12±0.01 | 0.21±0.02 | 0.34±0.05 | 0.35±0.05 | 0.24±0.02 | 0.28±0.01 | *0.22* | *0.25* |
| Ornithine | 1.35±0.18 | 0.31±0.05 | 0.52±0.04 | 0.35±0.02 | 2.76±0.26 | 0.31±0.05 | 3.66±0.41 | 0.34±0.01 | *2.07* | *0.33* |
| Phenylalanine | 0.12±0.011 | 0.28±0.06 | 0.16±0.02 | 0.30±0.03 | 0.12±0.02 | 0.63±0.10 | 0.17±0.01 | 0.42±0.04 | *0.14* | *0.41* |
| Proline | 4.64±0.08 | 3.39±0.11 | 3.97±0.27 | 5.74±0.16 | 5.42±0.22 | 5.37±0.06 | 5.00±0.17 | 4.78±0.16 | *4.76* | *4.82* |
| Serine | 6.80±1.10 | 5.09±0.73 | 12.1±2.6 | 20.8±3.1 | 4.53±0.09 | 43.3±3.1 | 2.58±0.44 | 15.5±1.4 | *6.51* | *21.2* |
| Threonine | 16.2±1.6 | 21.7±3.8 | 49.1±6.6 | 56.4±6.5 | 31.6±5.6 | 65.5±10.7 | 64.4±5.1 | 81.5±3.8 | *40.3* | *56.3* |
| Tryptophan | 0.21±0.04 | 0.08±0.01 | 0.37±0.06 | 0.46±0.03 | 0.30±0.04 | 0.74±0.11 | 0.35±0.03 | 0.45±0.02 | *0.31* | *0.43* |
| Tyrosine | 8.25±0.98 | 0.87±0.24 | 5.51±0.62 | 2.03±0.28 | 10.8±1.8 | 6.20±0.83 | 11.7±0.9 | 2.12±0.04 | *9.06* | *2.81* |
| Valine | 2.63±0.22 | 1.01±0.14 | 1.76±0.18 | 1.71±0.16 | 3.47±0.50 | 2.09±0.28 | 3.70±0.24 | 1.82±0.07 | *2.89* | *1.66* |
| BCAAs | 4.12±0.44 | 2.01±0.28 | 2.28±0.22 | 3.26±0.30 | 4.59±0.63 | 4.43±0.79 | 4.93±0.39 | 3.22±0.13 | *3.98* | *3.23* |
| Minor AA | 21.1±2.5 | 6.71±1.05 | 12.3±1.4 | 10.7±1.1 | 26.1±3.5 | 20.7±2.0 | 28.4±2.3 | 11.5±0.4 | *22.1* | *12.4* |
| Total AA | 581±49 | 85.6±12.5 | 374±40 | 168±18 | 758±99 | 224±27 | 743±49 | 194±8 | *614* | *168* |

**Supplemental table 3**: Chlorophyll *a*, *b*, carotenoids, polyphenols (in µg g^-1^ DW), soluble proteins, starch, glucose, fructose, sucrose (in mg g^-1^ DW), and free amino acid content (in µmol g^-1^ DW) in leaves and tuberous roots of *Ranunculus asiaticus* L. plants hybrid MBO at pre germination, leaf rosette and flowering stage; growth after pre-germination in a cold greenhouse under four photoperiodic lighting treatments: NL, NL + photoperiodic lighting with fluorescence light (FL), NL + photoperiodic lighting with LEDs R:FR 3:1 (R:FR 3:1), NL + photoperiodic lighting with LEDs R:FR 1:3 (R:FR 1:3). Non significance or significance differences at *p* ≤ 0.05 are indicated as: ns and * respectively.

| MBO | | | | | | | | |
| --- | --- | --- | --- | --- | --- | --- | --- | --- |
|  | Lighting treatment (L) | | | | Phenological stage (P) | | L X P | |
|  | Leaf rosette | | Flowering | |  |  |  |  |
| Plant organ | Tuberous roots | Leaves | Tuberous roots | Leaves | Tuberous roots | Leaves | Tuberous roots | Leaves |
| Chl *a* |  | ns |  | ns |  | * |  | * |
| Chl *b* |  | ns |  | ns |  | * |  | * |
| Carotenoids |  | ns |  | ns |  | * |  | * |
| Soluble proteins | ns | * | * | * | * | * | * | * |
| Starch | ns | ns | * | * | * | ns | * | * |
| Glucose | * | * | * | * | * | * | * | * |
| Fructose | * | * | * | * | * | ns | * | * |
| Sucrose | * | * | * | * | * | * | * | * |
| Polyphenols | * | ns | * | ns | * | ns | * | * |
| Alanine | * | * | * | * | * | * | * | * |
| Arginine | * | * | * | * | * | ns | * | * |
| Asparagine | ns | * | * | * | * | * | * | * |
| Aspartate | * | ns | * | ns | * | * | * | * |
| GABA | * | * | Ns | * | * | * | * | * |
| Glutamate | * | ns | * | ns | * | * | * | * |
| Glutamine | * | * | * | * | * | * | * | * |
| Glycine | ns | * | * | * | * | * | * | * |
| Histidine | * | * | * | * | * | * | * | * |
| Isoleucine | * | * | * | ns | * | ns | * | * |
| Leucine | * | * | * | * | * | * | * | * |
| Lysine | * | * | * | * | * | * | * | * |
| MEA | * | * | * | * | * | ns | * | * |
| Methionine | * | * | * | * | * | * | * | * |
| Ornithine | * | ns | * | ns | * | ns | * | ns |
| Phenylalanine | * | * | Ns | * | * | * | * | * |
| Proline | * | * | * | * | * | ns | * | * |
| Serine | * | ns | * | * | * | * | * | * |
| Threonine | * | * | * | * | * | * | * | * |
| Tryptophan | * | * | Ns | * | * | ns | * | * |
| Tyrosine | * | * | * | * | * | * | * | * |
| Valine | * | * | * | * | * | ns | * | * |
| BCAAs | * | * | * | * | * | ns | * | * |
| Minor AA | * | * | * | * | * | ns | * | * |
| Total AA | ns | ns | * | * | * | * | * | * |

**Supplemental table 4**: Chlorophyll *a*, *b*, carotenoids, polyphenols (in µg g^-1^ DW), soluble proteins, starch, glucose, fructose, sucrose (in mg g^-1^ DW), and free amino acid content (in µmol g^-1^ DW) in leaves and tuberous roots of *Ranunculus asiaticus* L. plants hybrid MDR at pre germination and leaf rosette stage; growth after pre-germination in a cold greenhouse under four photoperiodic lighting treatments: NL, NL + photoperiodic lighting with fluorescence light (FL), NL + photoperiodic lighting with LEDs R:FR 3:1 (R:FR 3:1), NL + photoperiodic lighting with LEDs R:FR 1:3 (R:FR 1:3). Mean values ± SE; n = 3.

|  | MDR | | | | | | | | | | |
| --- | --- | --- | --- | --- | --- | --- | --- | --- | --- | --- | --- |
|  |  | NL | | FL | | R:FR 3:1 | | R:FR 1:3 | |  |  |
| Phenological stage | Pre-Germination | Leaf rosette | | | | | | | | | |
| Plant organ | Tuberous roots | Tuberous roots | Leaves | Tuberous roots | Leaves | Tuberous roots | Leaves | Tuberous roots | Leaves | *Mean (Tuberous roots)* | *Mean (Leaves)* |
| Chl *a* |  |  | 0.12±0.01 |  | 0.13±0.01 |  | 0.12±0.01 |  | 0.19±0.01 |  | *0.14* |
| Chl *b* |  |  | 0.04±0.00 |  | 0.04±0.00 |  | 0.03±0.00 |  | 0.05±0.00 |  | *0.04* |
| Carotenoids |  |  | 0.03±0.00 |  | 0.04±0.00 |  | 0.03±0.00 |  | 0.05±0.00 |  | *0.04* |
| Soluble proteins | 41.4±1.8 | 17.0±0.3 | 27.6±1.5 | 19.3±0.8 | 29.7±1.2 | 25.4±0.7 | 23.9±1.8 | 23.7±0.9 | 32.4±1.1 | *21.3* | *28.4* |
| Starch | 17.5±0.5 | 8.82±0.18 | 11.0±0.5 | 11.3±0.6 | 15.1±0.8 | 11.7±0.2 | 14.6±1.5 | 7.41±0.40 | 12.7±0.5 | *9.82* | *13.4* |
| Glucose | 23.5±0.8 | 26.0±0.9 | 39.1±0.8 | 24.6±1.0 | 42.2±0.6 | 20.8±0.7 | 36.2±0.7 | 17.7±0.3 | 37.3±0.2 | *22.2* | *38.7* |
| Fructose | 29.6±0.5 | 12.9±0.4 | 26.3±0.1 | 14.9±0.6 | 26.0±0.3 | 16.8±0.4 | 22.2±0.2 | 9.85±0.21 | 25.8±0.4 | *13.6* | *25.1* |
| Sucrose | 12.7±0.2 | 5.52±0.28 | 6.28±0.08 | 6.81±0.26 | 6.28±0.04 | 7.55±0.21 | 5.63±0.14 | 4.02±0.11 | 6.55±0.22 | *5.97* | *6.19* |
| Polyphenols | 5.37±0.25 | 10.2±0.1 | 11.3±0.3 | 10.4±0.1 | 11.6±0.3 | 10.3±0.1 | 10.3±0.6 | 10.0±0.3 | 8.07±0.09 | *10.2* | *10.3* |
| Alanine | 4.30±0.57 | 2.60±0.399 | 5.00±0.37 | 3.45±0.30 | 4.60±0.76 | 20.4±1.9 | 2.54±0.08 | 2.07±0.07 | 6.03±0.90 | *7.14* | *4.54* |
| Arginine | 17.0±2.6 | 3.82±0.25 | 3.77±0.38 | 10.3±1.07 | 4.48±0.50 | 11.9±1.7 | 3.65±0.10 | 3.58±0.37 | 3.13±0.43 | *7.38* | *3.76* |
| Asparagine | 24.3±3.0 | 72.3±11.2 | 21.0±1.5 | 245±25 | 13.8±1.4 | 274±31 | 6.45±0.29 | 156±8 | 8.33±1.21 | *187* | *12.4* |
| Aspartate | 1.52±0.23 | 3.63±0.55 | 8.90±0.60 | 3.47±0.43 | 6.77±0.64 | 3.94±0.36 | 6.75±0.44 | 2.28±0.19 | 5.80±0.72 | *3.33* | *7.05* |
| GABA | 2.13±0.33 | 2.80±0.18 | 5.73±0.46 | 2.17±0.24 | 9.06±0.85 | 21.0±2.4 | 2.34±0.12 | 1.13±0.05 | 4.56±0.75 | *6.77* | *5.42* |
| Glutamate | 17.3±2.6 | 14.9±2.2 | 38.9±3.0 | 20.3±1.9 | 24.1±2.1 | 18.4±2.0 | 22.9±1.2 | 9.19±0.40 | 26.9±3.8 | *15.7* | *28.2* |
| Glutamine | 117±17 | 52.5±7.9 | 26.5±2.0 | 73.5±15.1 | 17.3±1.6 | 75.6±8.3 | 15.5±0.6 | 23.5±1.2 | 18.1±3.3 | *56.3* | *19.3* |
| Glycine | 6.07±0.98 | 7.37±1.21 | 0.85±0.14 | 10.2±1.7 | 0.77±0.08 | 14.6±0.8 | 0.24±0.03 | 7.41±0.22 | 0.94±0.14 | *9.89* | *0.70* |
| Histidine | 0.81±0.12 | 1.11±0.15 | 0.26±0.03 | 1.88±0.15 | 0.24±0.00 | 1.91±0.22 | 0.06±0.01 | 1.14±0.06 | 0.12±0.02 | *1.51* | *0.17* |
| Isoleucine | 10.7±1.6 | 1.26±0.21 | 1.21±0.08 | 3.12±0.24 | 1.00±0.08 | 3.37±0.33 | 1.12±0.05 | 1.12±0.04 | 0.79±0.10 | *2.22* | *1.03* |
| Leucine | 5.11±0.73 | 0.67±0.11 | 1.10±0.19 | 1.83±0.18 | 1.94±0.34 | 1.66±0.16 | 1.33±0.04 | 0.78±0.03 | 1.18±0.15 | *1.24* | *1.39* |
| Lysine | 1.23±0.07 | 2.05±0.134 | 1.24±0.21 | 3.30±0.30 | 2.59±0.22 | 2.22±0.30 | 2.08±0.06 | 2.28±0.16 | 1.54±0.24 | *2.46* | *1.86* |
| MEA | 6.21±0.36 | 2.22±0.16 | 8.43±0.65 | 2.43±0.31 | 9.14±0.89 | 2.33±0.32 | 6.07±0.26 | 1.55±0.10 | 7.86±1.32 | *2.13* | *7.87* |
| Methionine | 0.41±0.06 | 0.09±0.01 | 0.15±0.02 | 0.15±0.01 | 0.26±0.02 | 0.16±0.02 | 0.13±0.01 | 0.09±0.00 | 0.17±0.02 | *0.12* | *0.18* |
| Ornithine | 0.91±0.07 | 0.41±0.05 | 1.15±0.10 | 1.24±0.22 | 0.41±0.02 | 0.69±0.03 | 0.38±0.01 | 0.40±0.00 | 0.49±0.09 | *0.68* | *0.61* |
| Phenylalanine | 1.70±0.09 | 1.41±0.22 | 1.24±0.10 | 2.58±0.29 | 1.16±0.09 | 2.41±0.25 | 0.89±0.02 | 1.49±0.11 | 0.99±0.16 | *1.97* | *1.07* |
| Proline | 5.96±0.22 | 3.63±0.17 | 4.66±0.10 | 4.24±0.08 | 3.36±0.06 | 6.13±0.05 | 3.42±0.10 | 5.51±0.08 | 4.32±0.22 | *4.88* | *3.94* |
| Serine | 1.79±0.18 | 3.64±0.51 | 17.7±1.7 | 10.2±0.8 | 24.5±2.5 | 8.36±1.32 | 7.73±0.46 | 3.64±0.40 | 29.1±3.8 | *6.47* | *19.7* |
| Threonine | 9.88±1.45 | 5.10±0.31 | 19.6±1.7 | 9.23±1.07 | 15.8±1.7 | 7.53±1.05 | 28.5±1.2 | 4.76±0.23 | 1.80±0.28 | *6.65* | *16.4* |
| Tryptophan | 1.49±0.24 | 1.64±0.26 | 0.53±0.06 | 4.17±0.36 | 0.49±0.09 | 4.67±0.48 | 0.35±0.01 | 1.76±0.07 | 0.27±0.04 | *3.06* | *0.41* |
| Tyrosine | 7.22±1.05 | 2.54±0.41 | 1.19±0.09 | 4.18±0.35 | 0.86±0.07 | 4.83±0.48 | 0.75±0.02 | 1.77±0.06 | 0.67±0.10 | *3.33* | *0.87* |
| Valine | 16.6±2.4 | 2.69±0.40 | 1.39±0.09 | 7.37±0.59 | 1.34±0.11 | 6.93±0.67 | 1.32±0.06 | 2.84±0.12 | 1.17±0.15 | *4.96* | *1.30* |
| BCAAs | 32.5±4.7 | 4.62±0.72 | 3.69±0.35 | 12.3±0.8 | 4.28±0.52 | 12.0±1.15 | 3.77±0.14 | 4.75±0.18 | 3.14±0.39 | *8.41* | *3.72* |
| Minor AA | 62.3±9.0 | 17.3±2.2 | 12.1±1.2 | 38.8±2.8 | 14.3±1.5 | 40.0±4.6 | 11.7±0.3 | 16.9±1.0 | 10.0±1.4 | *28.2* | *12.0* |
| Total AA | 259±36 | 188±27 | 170±13 | 424±47 | 144±14 | 493±54 | 115±4 | 234±11 | 124±16 | *335* | *138* |

**Supplemental table 5**: Chlorophyll *a*, *b*, carotenoids, polyphenols (in µg g^-1^ DW), soluble proteins, starch, glucose, fructose, sucrose (in mg g^-1^ DW), and free amino acid content (in µmol g^-1^ DW) in leaves and tuberous roots of *Ranunculus asiaticus* L. plants hybrid MDR at flowering stage; growth after pre-germination in a cold greenhouse under four photoperiodic lighting treatments: NL, NL + photoperiodic lighting with fluorescence light (FL), NL + photoperiodic lighting with LEDs R:FR 3:1 (R:FR 3:1), NL + photoperiodic lighting with LEDs R:FR 1:3 (R:FR 1:3). Mean values ± SE; n = 3.

|  | MDR | | | | | | | | | |
| --- | --- | --- | --- | --- | --- | --- | --- | --- | --- | --- |
|  | NL | | FL | | R:FR 3:1 | | R:FR 1:3 | |  |  |
| Phenological stage | Flowering | | | | | | | | | |
| Plant organ | Tuberous roots | Leaves | Tuberous roots | Leaves | Tuberous roots | Leaves | Tuberous roots | Leaves | *Mean (Tuberous roots)* | *Mean (Leaves)* |
| Chl *a* |  | 0.11±0.00 |  | 0.12±0.01 |  | 0.15±0.02 |  | 0.07±0.01 |  | *0.11* |
| Chl *b* |  | 0.03±0.00 |  | 0.04±0.00 |  | 0.05±0.01 |  | 0.02±0.00 |  | *0.03* |
| Carotenoids |  | 0.03±0.00 |  | 0.04±0.00 |  | 0.04±0.00 |  | 0.02±0.00 |  | *0.03* |
| Soluble proteins | 31.2±1.6 | 30.4±0.3 | 31.1±1.0 | 25.9±0.8 | 28.8±1.7 | 24.5±1.1 | 28.7±2.0 | 19.6±0.4 | *29.9* | *25.1* |
| Starch | 30.0±1.7 | 11.2±1.2 | 31.1±0.8 | 15.8±0.3 | 32.3±3.1 | 13.8±0.9 | 31.9±2.9 | 15.1±0.4 | *31.4* | *14.0* |
| Glucose | 28.0±1.1 | 36.8±0.6 | 17.2±0.5 | 34.8±0.4 | 18.3±0.9 | 26.3±0.4 | 16.1±2.5 | 33.6±2.6 | *19.9* | *32.9* |
| Fructose | 20.9±0.1 | 24.4±0.4 | 21.7±0.6 | 26.4±0.2 | 21.2±0.3 | 27.6±0.4 | 19.4±1.1 | 27.2±0.9 | *20.8* | *26.4* |
| Sucrose | 9.93±0.15 | 6.45±0.15 | 10.4±0.3 | 7.68±0.07 | 9.95±0.03 | 8.08±0.02 | 9.12±0.60 | 6.55±0.22 | *9.85* | *7.19* |
| Polyphenols | 14.7±0.5 | 12.0±0.3 | 7.53±0.05 | 11.4±0.0 | 10.3±0.5 | 10.9±0.1 | 10.8±1.3 | 13.7±0.7 | *10.8* | *12.0* |
| Alanine | 5.09±0.55 | 4.64±0.29 | 4.21±0.24 | 2.89±0.44 | 2.09±0.17 | 4.50±0.37 | 4.41±0.50 | 4.02±0.69 | *3.95* | *4.01* |
| Arginine | 4.63±0.56 | 2.53±0.18 | 3.24±0.15 | 1.70±0.24 | 2.08±0.04 | 2.35±0.27 | 3.60±0.33 | 1.83±0.23 | *3.39* | *2.10* |
| Asparagine | 226±29 | 3.96±0.27 | 59.0±4.2 | 1.51±0.24 | 70.1±12.2 | 2.48±0.43 | 113±14 | 2.18±0.15 | *117* | *2.53* |
| Aspartate | 6.52±0.80 | 4.27±0.20 | 8.67±0.60 | 4.27±0.60 | 4.17±0.35 | 4.79±0.62 | 8.84±0.34 | 3.48±0.61 | *7.05* | *4.20* |
| GABA | 4.19±0.62 | 3.38±0.29 | 5.44±0.28 | 1.12±0.06 | 1.83±0.16 | 1.02±0.14 | 3.95±0.68 | 2.12±0.18 | *3.85* | *1.91* |
| Glutamate | 43.0±4.8 | 13.6±0.9 | 44.7±2.7 | 16.1±2.3 | 20.5±2.3 | 13.2±2.3 | 42.7±5.0 | 13.4±2.4 | *37.7* | *14.1* |
| Glutamine | 190±22.7 | 20.7±1.4 | 85.2±5.0 | 6.88±0.98 | 37.3±3.3 | 14.9±1.9 | 109±14 | 9.18±1.60 | *105* | *12.9* |
| Glycine | 27.2±1.6 | 0.19±0.02 | 9.35±0.67 | 0.27±0.01 | 4.61±0.11 | 0.43±0.06 | 12.8±2.1 | 0.15±0.02 | *13.5* | *0.26* |
| Histidine | 0.65±0.07 | 0.39±0.03 | 0.33±0.02 | 0.38±0.05 | 0.15±0.01 | 0.13±0.02 | 0.41±0.04 | 0.12±0.02 | *0.39* | *0.25* |
| Isoleucine | 0.68±0.07 | 0.48±0.03 | 0.43±0.02 | 0.32±0.04 | 0.34±0.03 | 0.52±0.06 | 0.50±0.04 | 0.32±0.05 | *0.49* | *0.41* |
| Leucine | 0.40±0.06 | 0.54±0.04 | 0.36±0.04 | 0.41±0.06 | 0.25±0.05 | 0.44±0.05 | 0.45±0.03 | 0.54±0.09 | *0.37* | *0.48* |
| Lysine | 3.25±0.53 | 0.36±0.03 | 3.30±0.13 | 0.33±0.06 | 2.98±0.23 | 0.45±0.05 | 3.36±0.09 | 0.35±0.03 | *3.22* | *0.38* |
| MEA | 1.84±0.27 | 2.72±0.24 | 2.46±0.24 | 2.94±0.18 | 0.92±0.15 | 3.95±0.55 | 2.23±0.20 | 2.81±0.25 | *1.86* | *3.11* |
| Methionine | 0.17±0.02 | 0.17±0.01 | 0.30±0.02 | 0.10±0.02 | 0.13±0.02 | 0.13±0.02 | 0.20±0.03 | 0.17±0.03 | *0.20* | *0.14* |
| Ornithine | 3.38±0.46 | 0.18±0.02 | 2.69±0.11 | 0.12±0.02 | 1.50±0.12 | 0.11±0.01 | 2.20±0.16 | 0.10±0.01 | *2.44* | *0.13* |
| Phenylalanine | 0.21±0.02 | 0.32±0.02 | 0.12±0.01 | 0.28±0.04 | 0.06±0.01 | 0.32±0.06 | 0.13±0.01 | 0.33±0.06 | *0.13* | *0.31* |
| Proline | 4.19±0.12 | 4.71±0.06 | 2.35±0.08 | 1.98±0.06 | 3.27±0.13 | 2.91±0.06 | 2.97±0.13 | 3.06±0.12 | *3.20* | *3.16* |
| Serine | 1.32±0.15 | 8.73±0.63 | 0.69±0.05 | 4.81±0.83 | 0.49±0.08 | 8.12±1.37 | 0.86±0.09 | 7.52±0.61 | *0.84* | *7.30* |
| Threonine | 27.7±4.4 | 2.63±0.42 | 27.8±2.0 | 7.23±1.26 | 19.2±1.8 | 26.8±3.9 | 39.1±6.2 | 15.8±1.3 | *28.4* | *13.1* |
| Tryptophan | 0.15±0.03 | 0.07±0.00 | 0.18±0.02 | 0.19±0.02 | 0.09±0.00 | 0.13±0.01 | 0.17±0.02 | 0.07±0.00 | *0.15* | *0.11* |
| Tyrosine | 6.16±0.73 | 2.18±0.16 | 3.21±0.22 | 1.07±0.15 | 1.38±0.13 | 2.09±0.25 | 4.05±0.59 | 1.37±0.11 | *3.70* | *1.68* |
| Valine | 2.75±0.29 | 1.08±0.08 | 1.87±0.11 | 0.66±0.09 | 1.10±0.12 | 0.96±0.10 | 1.92±0.20 | 0.76±0.12 | *1.91* | *0.86* |
| BCAAs | 3.83±0.42 | 2.10±0.14 | 2.66±0.09 | 1.38±0.19 | 1.70±0.19 | 1.93±0.21 | 2.87±0.26 | 1.61±0.26 | *2.76* | *1.75* |
| Minor AA | 19.0±2.3 | 8.10±0.57 | 13.3±0.6 | 5.42±0.76 | 8.56±0.17 | 7.52±0.85 | 14.8±1.4 | 5.87±0.74 | *13.9* | *6.73* |
| Total AA | 559±67 | 77.7±5.2 | 265±16 | 55.5±7.6 | 175±8 | 90.7±11.3 | 357±43 | 69.7±8.4 | *339* | *73.4* |

**Supplemental table 6**: Chlorophyll *a*, *b*, carotenoids, polyphenols (in µg g^-1^ DW), soluble proteins, starch, glucose, fructose, sucrose (in mg g^-1^ DW), and free amino acid content (in µmol g^-1^ DW) in leaves and tuberous roots of *Ranunculus asiaticus* L. plants hybrid MDR at leaf rosette and flowering stage; growth after pre-germination in a cold greenhouse under four photoperiodic lighting treatments: NL, NL + photoperiodic lighting with fluorescence light (FL), NL + photoperiodic lighting with LEDs R:FR 3:1 (R:FR 3:1), NL + photoperiodic lighting with LEDs R:FR 1:3 (R:FR 1:3). Non-significant or significant differences at *p* ≤ 0.05 are indicated as: ns and * respectively.

| MDR | | | | | | | | |
| --- | --- | --- | --- | --- | --- | --- | --- | --- |
|  | Lighting treatment (L) | | | | Phenological stage | | L X P | |
|  | Leaf rosette | | Flowering | |  |  |  |  |
| Plant organ | Tuberous roots | Leaves | Tuberous roots | Leaves | Tuberous roots | Leaves | Tuberous roots | Leaves |
| Chl *a* |  | * |  | * |  | * |  | * |
| Chl *b* |  | * |  | * |  | ns |  | * |
| Carotenoids |  | * |  | * |  | ns |  | * |
| Soluble proteins | * | * | Ns | * | * | ns | * | * |
| Starch | * | * | Ns | * | * | ns | * | * |
| Glucose | * | * | * | * | * | * | * | * |
| Fructose | * | * | Ns | * | * | ns | * | * |
| Sucrose | * | * | Ns | * | * | * | * | * |
| Polyphenols | ns | * | * | * | * | * | * | * |
| Alanine | * | * | * | ns | * | ns | * | * |
| Arginine | * | ns | * | ns | * | * | * | * |
| Asparagine | * | * | * | * | * | * | * | * |
| Aspartate | ns | * | * | ns | * | * | * | * |
| GABA | * | * | * | * | * | * | * | * |
| Glutamate | * | * | * | ns | * | * | * | * |
| Glutamine | * | * | * | * | * | * | * | * |
| Glycine | * | * | * | * | * | * | * | * |
| Histidine | * | * | * | * | * | ns | * | * |
| Isoleucine | * | * | * | * | * | * | * | * |
| Leucine | * | ns | * | ns | * | * | * | * |
| Lysine | * | * | Ns | ns | * | * | * | * |
| MEA | ns | ns | * | ns | * | * | * | * |
| Methionine | * | * | * | ns | * | ns | * | * |
| Ornithine | * | * | * | * | * | * | * | * |
| Phenylalanine | * | ns | * | ns | * | * | * | * |
| Proline | * | * | * | * | * | * | * | * |
| Serine | * | * | * | ns | * | * | * | * |
| Threonine | * | * | * | * | * | ns | * | * |
| Tryptophan | * | * | * | * | * | * | * | * |
| Tyrosine | * | * | * | * | * | * | * | * |
| Valine | * | ns | * | ns | * | * | * | * |
| BCAAs | * | ns | * | ns | * | * | * | * |
| Minor AA | * | ns | * | ns | * | * | * | * |
| Total AA | * | ns | * | ns | ns | * | * | * |
